# Articular Cartilage Assessment Using Ultrashort Echo Time MRI: A Review

**DOI:** 10.3389/fendo.2022.892961

**Published:** 2022-05-26

**Authors:** Amir Masoud Afsahi, Sam Sedaghat, Dina Moazamian, Ghazaleh Afsahi, Jiyo S. Athertya, Hyungseok Jang, Ya-Jun Ma

**Affiliations:** ^1^Department of Radiology, University of California San Diego, San Diego, CA, United States; ^2^Research Service, Veterans Affairs San Diego Healthcare System, San Diego, CA, United States; ^3^Department of Biotechnology Research, BioSapien, San Diego, CA, United States

**Keywords:** ultrashort echo time, MRI, knee, cartilage, deep layer cartilage, calcified cartilage

## Abstract

Articular cartilage is a major component of the human knee joint which may be affected by a variety of degenerative mechanisms associated with joint pathologies and/or the aging process. Ultrashort echo time (UTE) sequences with a TE less than 100 µs are capable of detecting signals from both fast- and slow-relaxing water protons in cartilage. This allows comprehensive evaluation of all the cartilage layers, especially for the short T_2_ layers which include the deep and calcified zones. Several ultrashort echo time (UTE) techniques have recently been developed for both morphological imaging and quantitative cartilage assessment. This review article summarizes the current catalog techniques based on UTE Magnetic Resonance Imaging (MRI) that have been utilized for such purposes in the human knee joint, such as T_1_, ${\text{T}}_{2}^{*}$ , T_1ρ_, magnetization transfer (MT), double echo steady state (DESS), quantitative susceptibility mapping (QSM) and inversion recovery (IR). The contrast mechanisms as well as the advantages and disadvantages of these techniques are discussed.

## Introduction

Articular cartilage is one of the most important components of the human knee joint, distributing compressive loads and enabling low-friction motion (1). Medical imaging is of critical role in cartilage assessment for screening and treatment purposes (2, 3).

Magnetic resonance imaging (MRI) has long been a useful modality in the investigation of musculoskeletal (MSK) components, particularly in the case of evaluating articular cartilage in the human knee joint. Collagenous matrix is one of the primary components of cartilage, which has a higher structural order at the deeper layers of cartilage. Part of the water components in cartilage are tightly bound to the collagenous matrix (i.e., bound water). This bound water component typically has a much shorter T_2_ relative to the free water component due to its limited mobility. Deeper layers of cartilage consist of more bound water because of the denser collagen structures in these regions. As a result, cartilage has a relatively short T_2_ relaxation, especially when the collagen fiber orientates in parallel to the B_0_ field due to the magic angle effect (4). In general, T_2_ in tissues can be categorized into the four following groups: “super-short” (T_2_ <0.1 ms), “ultrashort” (0.1<T_2_<1 ms), “short” (1<T_2_<10 ms), and “long” (T_2_>10 ms) (5). While conventional MRI is oftentimes able to provide accurate morphological assessment of many musculoskeletal tissues throughout the body with high spatial resolution, in the case of articular cartilage, conventional MRI techniques such as the fast spin echo (FSE) sequence are, in fact, incapable of capturing this tissue’s short T_2_ signals due to the MRI sequences’ relatively long echo times (TEs) (>5 ms).

During the last two decades, a specific MRI approach termed ultrashort echo time (UTE) MRI has gained traction in the research sphere as a technique that is able to visualize otherwise difficult-to-image tissue structures such as those with short T_2_ and ${\text{T}}_{2}^{*}$ values and/or low water and proton content (4, 6). Since UTE MRI’s very first introduction in the 1980s (7) as an *in vivo* application for lung parenchymal imaging (8), the technique has been further developed to visualize an array of other short T_2_ species such as menisci and tendons (9). By using UTE MRI, rapidly decaying signals that would typically be lost in conventional MRI can be acquired after short radiofrequency (RF) excitation, as quickly as is possible by the hardware (TE < 100μs), before any major T_2_/ ${\text{T}}_{2}^{*}$ decay (10). Basic UTE, dual echo UTE with echo subtraction (11), inversion recovery UTE (IR-UTE) (12), dual-inversion recovery UTE (Dual-IR-UTE) (13, 14), UTE ${\text{T}}_{2}^{*}$ (15), UTE-T1ρ (16, 17), UTE with magnetization transfer (MT) (18–20), UTE with double echo steady state (DESS) (21, 22), and UTE with quantitative susceptibility mapping (QSM) (23) are among the primary UTE MRI techniques which have been developed for qualitative and quantitative imaging of articular cartilage.

**Figure 1:**
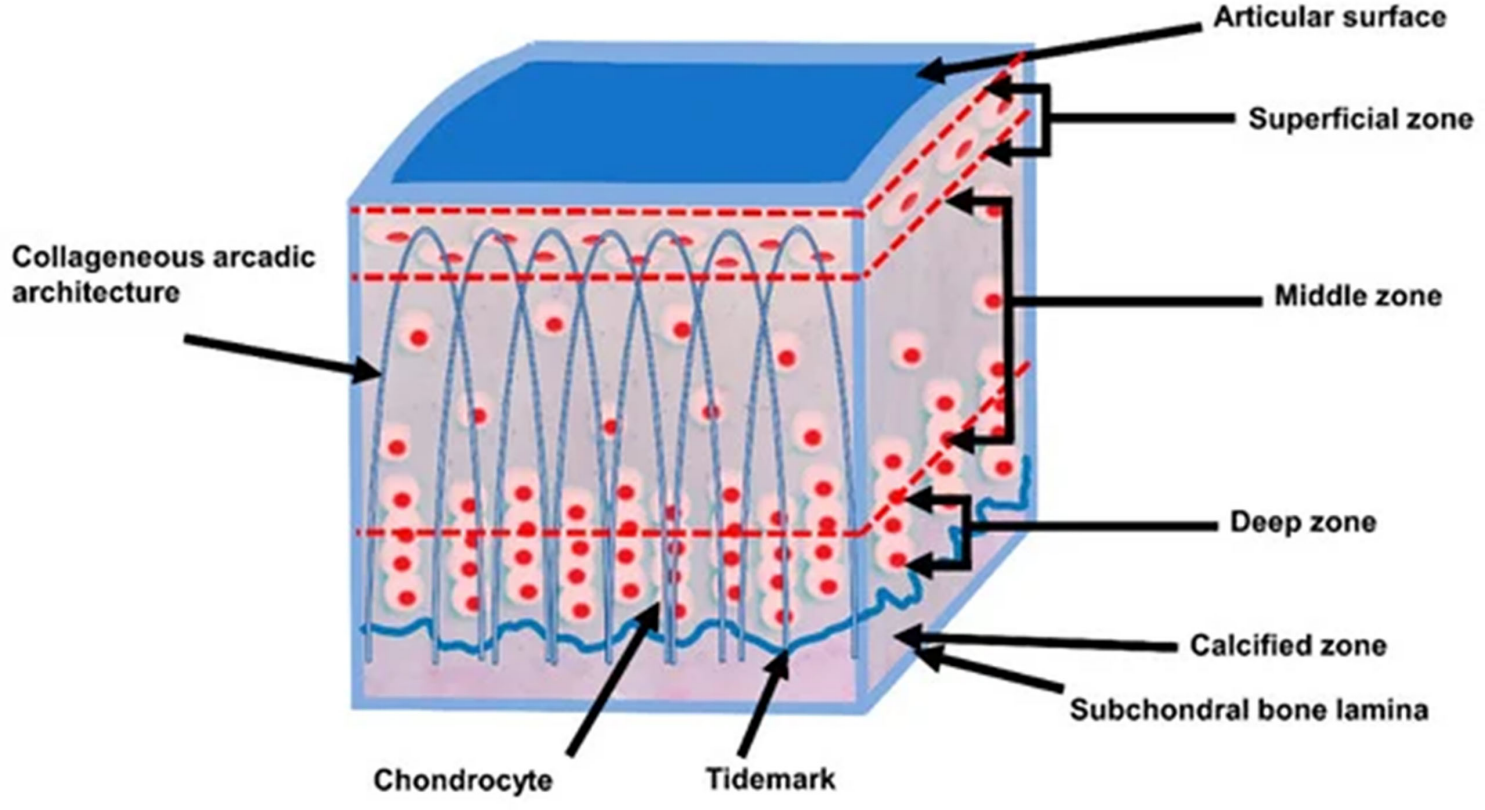
The schematic cross sectional layers of articular cartilage. Articular cartilage consists of four different layers, namely the superficial, middle, deep, and calcified layers. Modified, with permission under Creative Common Attribution License from Ref (24).

Articular cartilage consists of four different layers, namely the superficial, middle, deep, and calcified layers (see **Figure 1**). These layers are of increasing radiological significance as it has become known that they may play a role in the early pathogenesis of osteoarthritis (OA) (25). However, because of their short transverse relaxation times, these cartilaginous structures are not properly visualized when using conventional MR sequences (4). UTE MRI’s ability to capture these short transverse relaxation time tissues means that all the layers of cartilage, including both the short and long T_2_ layers, can be more accurately and comprehensively imaged and quantified for improved assessment of diseases such as osteoarthritis compared to conventional MRI sequences which can only image the long T_2_ layers of cartilage (26–28).

This study is a systematic review focused on the current development of UTE MRI techniques applied for articular cartilage evaluation. It is categorized based on different UTE techniques including basic UTE, various IR-UTE methods, UTE ${\text{T}}_{2}^{*}$ , UTE T_1_, UTE T_1ρ_, UTE MT, UTE DESS, and UTE QSM. **Table 1** presents a summary of the articles reviewed in this study. This study is an update of the current review literature focused on musculoskeletal tissues with short T_2_ values (4, 6) as these techniques continue to rapidly evolve and have been applied in numerous other studies.

**Table 1:** Summary of articles in UTE MRI of articular cartilage.

1st Author, year/(Ref #)	Study specimens/Population	MRI sequence/Field strength
**Du et al., 2010/ (** 29**)**	^¹^Healthy adult volunteers for tendons, ligaments, aponeuroses, ¹ meniscus sample; ² patellar samples for cartilage;	Basic 3D UTE with ^*^several short T_2_ contrasts**/**3T
		* ¹(3D dUTE); and ²(DIR-UTE)/3T
**Du et al., 2012/ (** 13**)**	Cadaveric patellae and phantoms	DIR, Saturation recovery for ${\text{T}}_{2}^{*}$ and T_1_; Spin-locking-prepared DIR UTE for T_1ρ_/3T
**Gatehouse et al., 2004/ (** 30**)**	Patients vs volunteers	^*^ UTE and gadodiamide, conventional FS, subtraction**/**1.5T
		^*^TE= 0.08
**Ma et al., 2019/ (** 31**)**	Healthy volunteers	^*^T_1_ mapping**/**3T
		^*^AFI**-**VFA method
**Wu et al., 2019/ (** 32**)**	Cadaveric human knees and volunteers	^*^3D-UTE quantitative techniques**/**3T
		^*^T_1_, ${\text{T}}_{2}^{*}$ , AdiabT_1ρ_, MTR, and MT modeling
**Wan et al., 2020/ (** 33**)**	Human cadaveric whole knee from donors	^*^3D-UTE quantitative techniques and extended spiral sampling**/**3T
		^*^ ${\text{T}}_{2}^{*}$ , T_1_, AdiabT_1ρ_, MTR, and MMF
**Yang et al., 2020/ (** 19**)**	Degenerative anterolateral condyles of total knee arthroplasty patients	MT**/**3T
**Namiranian et al., 2020/ (** 34**)**	Tibio-femoral cartilages	^*^3D UTE quantitative/3T
		^*^MT (MTR, MMF, T_2_mm), AdiabT_1ρ_, T_1ρ_, ${\text{T}}_{2}^{*}$ mapping
**Foreman et al., 2019/ (** 35**)**	Type 2 Diabetics vs Non-diabetics	${\text{T}}_{2}^{*}$ mapping**/**3T
**Liu et al., 2019/ (** 26**)**	Cadaveric knee vs healthy humans	${\text{T}}_{2}^{*}$ mapping**/**3T
**Drygalsky et al., 2019/ (** 36**)**	Haemophilia A and B patients	${\text{T}}_{2}^{*}$ mapping**/**3T
**Williams et al., 2018/ (** 15**)**	ACL reconstructed patients vs uninjured patients	${\text{T}}_{2}^{*}$ mapping**/**3T
		2D ${\text{T}}_{2}^{*}$ for MFC and 3D for MTP
**Williams et al., 2018/ (** 37**)**	ACLR patients vs healthy volunteers	${\text{T}}_{2}^{*}$ mapping**/**3T
**Williams et al., 2011/ (** 38**)**	Asymptomatic subjects	${\text{T}}_{2}^{*}$ mapping**/**3T
**Williams et al., 2010/ (** 39**)**	Osteochondral cores of human tibial plateau	${\text{T}}_{2}^{*}$ mapping**/**3T
**Titchenal et al., 2018/ (** 40**)**	ACLR patients vs uninjured volunteers	${\text{T}}_{2}^{*}$ Mapping**/**3T
**Shao et al., 2016/ (** 41**)**	Cadaveric patellae	^*^Bicomponent ${\text{T}}_{2}^{*}$ **/**3T
		^*^Short and long ${\text{T}}_{2}^{*}$ values and fractions
**Chu et al., 2014/ (** 42**)**	ACLR patients vs uninjured volunteers	${\text{T}}_{2}^{*}$ mapping/3T
**Du et al., 2008/ (** 43**)**	Healthy volunteers	SUTE**/**1.5T
**Goto et al., 2012/ (** 44**)**	Healthy volunteers	UTE with Spiral acquisition/1.5T
**Van Dyck et al., 2015/ (** 45**)**	Healthy volunteers vs patients with clinical suspicion of knee cartilage abnormality	3D-UTE**/**3T


**Du et al., 2009/ (** 46**)**	Cadaveric samples vs human volunteers	UTESI**/**3T
**Chang G. et al., 2012/ (** 47**)**	Patients with cartilage restorative surgery	*3D-Na-UTE/7T
		*Without IR vs with IR to suppress Na signal of free fluid
**Larson et al., 2016/ (** 48**)**	Healthy volunteers	UTE vs ZTE**/**7T
**Ma et al., 2018/ (** 17**)**	*Ex vivo* human knees vs healthy volunteers	*3D UTE/3T
		*AdiabT_1ρ_
**Lee et al., 2014/ (** 49**)**	Normal MRI patients	*3D UTE**/**3T
		*Weighted subtraction
**Ma et al., 2019/ (** 50**)**	Phantom and *ex vivo* cartilage, meniscus	*3D UTE**/**3T
		*Acido CEST
**Qian et al., 2012/ (** 51**)**	Asymptomatic humans vs injured ACL patients	UTE with AWSOS sequence/3T
**Hananouchi et al., 2021/ (** 52**)**	Cadaveric patellar cartilages	3D UTE MT and ${\text{T}}_{2}^{*}$ /3T
**Qian et al., 2010/ (** 53**)**	Explants of cadaveric human tibial plateau cartilage, an explant of total knee arthroplasty	multi-component ${\text{T}}_{2}^{*}$ mapping and UTE/3T
**Pauli et al., 2012/ (** 54**)**	Human patella	2D UTE bicomponent/3T
		semiquantitative histopathologic and polarized light microscopic (PLM) assessments
**Du et al., 2012/ (** 55**)**	Goat ACL, bovine Achilles tendons, cadaveric human menisci, cadaveric human patellae, bovine cortical bone	2D UTE bicomponent ${\text{T}}_{2}^{*}$ /3T
**Jang et al., 2019/ (** 56**)**	Human cadaveric knee joints vs knee joints of healthy volunteers	2D UTE and single scan RHE for rapid bicomponent ${\text{T}}_{2}^{*}$ analysis/3T
**Jang et al., 2021/ (** 22**)**	Healthy volunteers vs OA patients	UTE-Cones-DESS for high contrast;
		(1p-Dixon)- based for fat suppression/3T
**Wu et al., 2020/ (** 57**)**	Human patellar samples	3D UTE Cones‐AdiabT_1ρ_/3T
		For comparison: 3D UTE Cones‐CW‐T_1ρ_ and Cones‐ ${\text{T}}_{2}^{*}$
**Jerban et al., 2020/ (** 58**)**	Young knee joints	AFI-VTR-based 3D UTE-Cones sequence for T_1_ measurement; 3D UTE-Cones-MT sequence for UTE-MT modeling/3T
**Xue et al., 2021/ (** 20**)**	Old volunteers with and without OA vs young healthy volunteers	UTE-MT sequence/3T
**Chen et al., 2022/ (** 59**)**	Knee cartilage samples and whole cadaveric knee specimens	*Quantitative 3D UTE with and without FatSat/3T
		*T_1ρ_, ${\text{T}}_{2}^{*}$ , and MT
**High et al., 2019/ (** 60**)**	Phantoms and patients with chronic knee pain	AcidoCEST 3D UTE/3T

## Basic UTE

**Figure 2:**
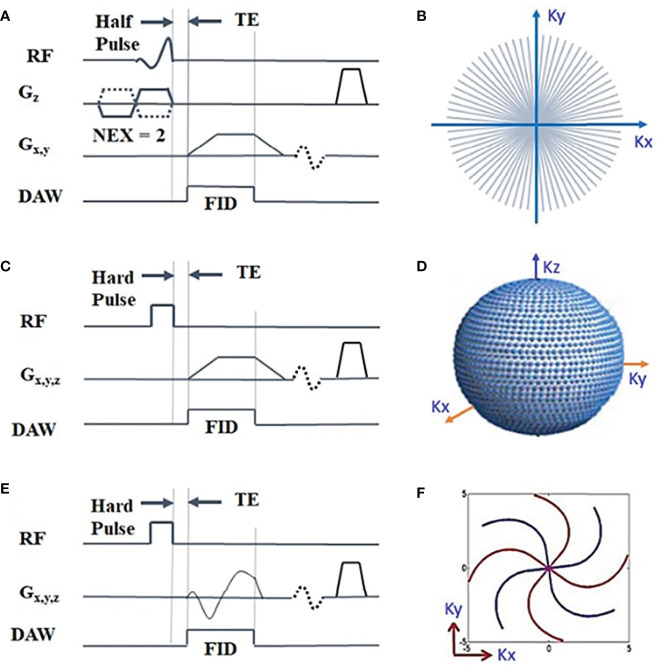
Three representative UTE pulse sequence diagrams: 2D UTE sequence with a slice-selective half radiofrequency (RF) pulse excitation followed by 2D radial ramp sampling **(A)** (4), and 3D UTE with a short hard pulse excitation followed by 3D radial ramp sampling **(C)** (4) or by twisted radial trajectories with conical view ordering (4, 31) (33, 62) **(E)**. The k-space sampling patterns are shown in **(B, D, F)**, respectively. The data acquisition window (DAW) covers part of the free induction decay (FID) before the short T_2_ transverse magnetization decays to near zero. Modified, with permission from Refs (4, 31).

Basic UTE, or UTE without any preparation pulses, has mainly been employed for morphological imaging of the articular cartilage. However, by using UTE images at varying echo times (TEs) or at varying repetition times (TRs) and flip angles (FAs), basic UTE has been used to measure cartilage ${\text{T}}_{2}^{*}$ (39, 61) and cartilage T1 (4, 29, 31), respectively. Basic UTE can also be performed with different readout trajectories. **Figure 2** illustrates three representative UTE pulse sequence diagrams. In UTE sequences, a short rectangular or half soft pulse is employed for signal excitation. Ramp sampling and fast transmit/receive switching strategy are utilized to minimize TE for the excited fid signals. Non-Cartesian k-space trajectories, such as radial and spiral, are the most commonly used spatial encoding patterns.

### Basic UTE for Morphological Imaging

**Figure 3:**
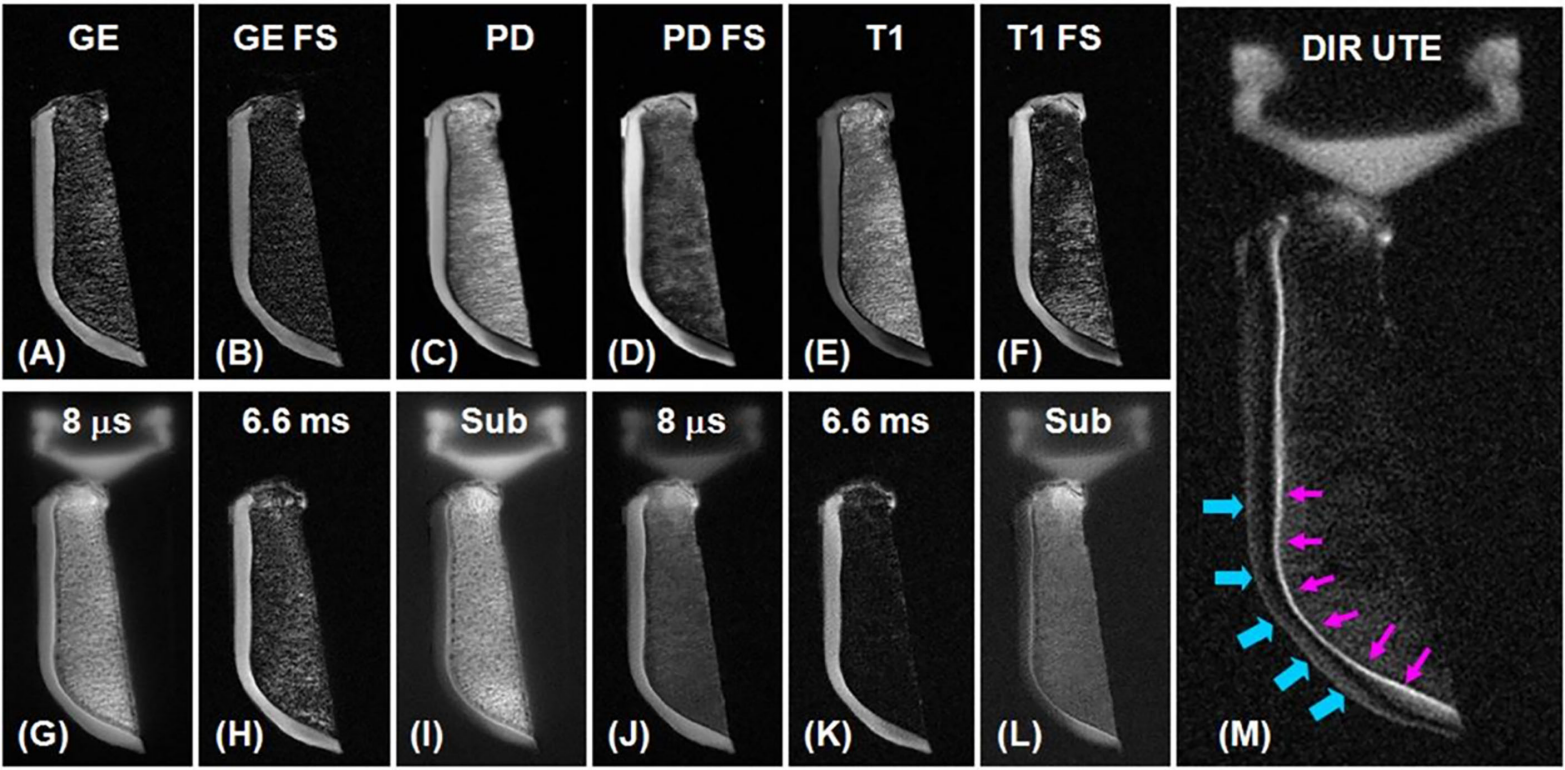
Axial imaging of a slice of patella with clinical gradient echo sequence (GE) **(A)**, GE with fat saturation (FS) **(B)**, proton density-weighted fast spin echo (PD FSE) **(C)**, PD FSE with FS **(D)**, T_1_ FSE **(E)**, T_1_ FSE with FS **(F)**, conventional UTE with a TE of 8 μs **(G)** and 6.6 ms **(H)**, subtraction of the second from the first echo **(I)**, fat-saturated UTE with a TE of 8 μs **(J)** and 6.6 ms **(K)** followed by corresponding later echo subtraction **(L)**, and dual inversion recovery (DIR) UTE **(M)**. Clinical GE and spin echo (SE)-FSE sequences do not show signal from deep radial and calcified cartilage layers, which are brightly visualized in UTE sequences. There is limited contrast between not only the deep and superficial layers of cartilage, but also between the cartilage layers and bone marrow fat. The DIR UTE image illustrates the deep radial and calcified cartilage layers with high contrast (pink arrows) and with good signal suppression from the superficial cartilage layers and fat. There is some residual signal from the superficial cartilage layers as a result of T_1_ variations (imperfect nulling). Modified, with permission from Ref (64).

One of the very first *in vivo* studies to apply UTE sequences in various parts of the knee, including articular cartilage, was conducted by Gatehouse et al., who used UTE sequences with a TE of 0.08 ms to visualize knee tissues in 16 patients who had various knee injuries on a 1.5T scanner (30). Using gadodiamide enhancement, conventional fat suppression, and subtraction images, they were able to detect higher signals from the deepest (i.e., calcified) cartilage layers which have shorter T_1_s and T_2_s relative to the more superficial cartilage layers. Cartesian and radial k-space sampling were originally used for UTE signal acquisition (63), but 2D spiral sampling has since been developed to accelerate the UTE imaging of short T_2_ species (43). Du et al. combined half-pulse excitation and spiral sampling for UTE imaging of the deep radial and calcified layers of knee cartilage in a healthy volunteer at 1.5T field strength and obtained good contrast and high spatial resolution (43). **Figure 3** illustrates axial imaging of a slice of patella with clinical gradient echo sequence (GE) (A), GE with fat saturation (FS) (B), proton density- weighted fast spin echo (PD FSE) (C), PD FSE with FS (D), T_1_ FSE (E), T_1_ FSE with FS (F), and conventional UTE with a TE of 8 μs (G) and of 6.6 ms (H), subtraction of the second from the first echo (I), fat-saturated UTE with a TE of 8 μs (J) and of 6.6 ms (K) followed by corresponding later echo subtraction (L), and dual inversion recovery (DIR) UTE (M).

Other studies have also employed spiral UTE MRI for deep cartilage morphological imaging. For example, Goto et al. (44) used spiral UTE sequences on 5 healthy volunteers to evaluate UTE sensitivity in the visualization of deep calcified cartilage layers and found a sufficient contrast of deep and calcified cartilage layers from the UTE dual-echo subtracted images.

Other variations of the spiral UTE MRI imaging technique have also been developed to reduce scan time and to improve the signal-to-noise ratio (SNR), resolution, and short T_2_ contrast within the knee joint (51, 65–67). Fermat looped, orthogonally encoded trajectories (FLORET) are superior to 3D radial acquisition with regards to image quality, SNR, scan time, and off-resonance blurring for UTE data (65). 3D cones trajectories (66), stack of spirals trajectories (67), and acquisition-weighted stack of spirals (AWSOS) (51) are all variations on UTE acquisition trajectories that have been used successfully. For example, Qian et al. utilized the AWSOS technique on a 3T scanner for *in vivo* morphological imaging of short T_2_ tissues in the knee joint, including articular cartilage (51). Spiral trajectories applied in this study accelerated in-plane data acquisitions, making the approach much more time-efficient with a reduced scan time by a factor of 4-10 compared with that in Cartesian acquisitions at the same resolution. Employing AWSOS, higher in-plane resolution (0.28-0.14mm) was achieved, making the technique potentially useful for earlier detection of pathology. However, to achieve an SNR acceptable for resolution of 0.14mm, a quite large slice thickness of 3mm is required, which results in strong partial volume effect. A smaller thickness of <2mm, although optimal, suffers SNR reductions at 3T. Higher field strengths such as 7T are a promising solution, as they can accommodate smaller slice thicknesses with sufficiently high SNR (48).

In another morphological study for visualization of short T_2_ tissues of the knee joint with optimal contrast, Lee et al. (49) implemented weighted subtraction in 3D UTE imaging on a 3T scanner. They hypothesized that weighting subtraction with an optimal weighting factor would provide high positive contrast of short T_2_ tissues with adequate suppression of the surrounding long T_2_ tissues. The optimal weighting factor in this study was calculated by determining the SNR and contrast-to-noise ratio (CNR), then dividing SNR by CNR. With a weighting factor of 0.4, they obtained a high contrast-weighted subtraction image in a 67-year-old man with prior clinical diagnoses of osteoarthritis and chondromalacia (49).

### UTE ${\text{T}}_{2}^{*}$

**Figure 4:**
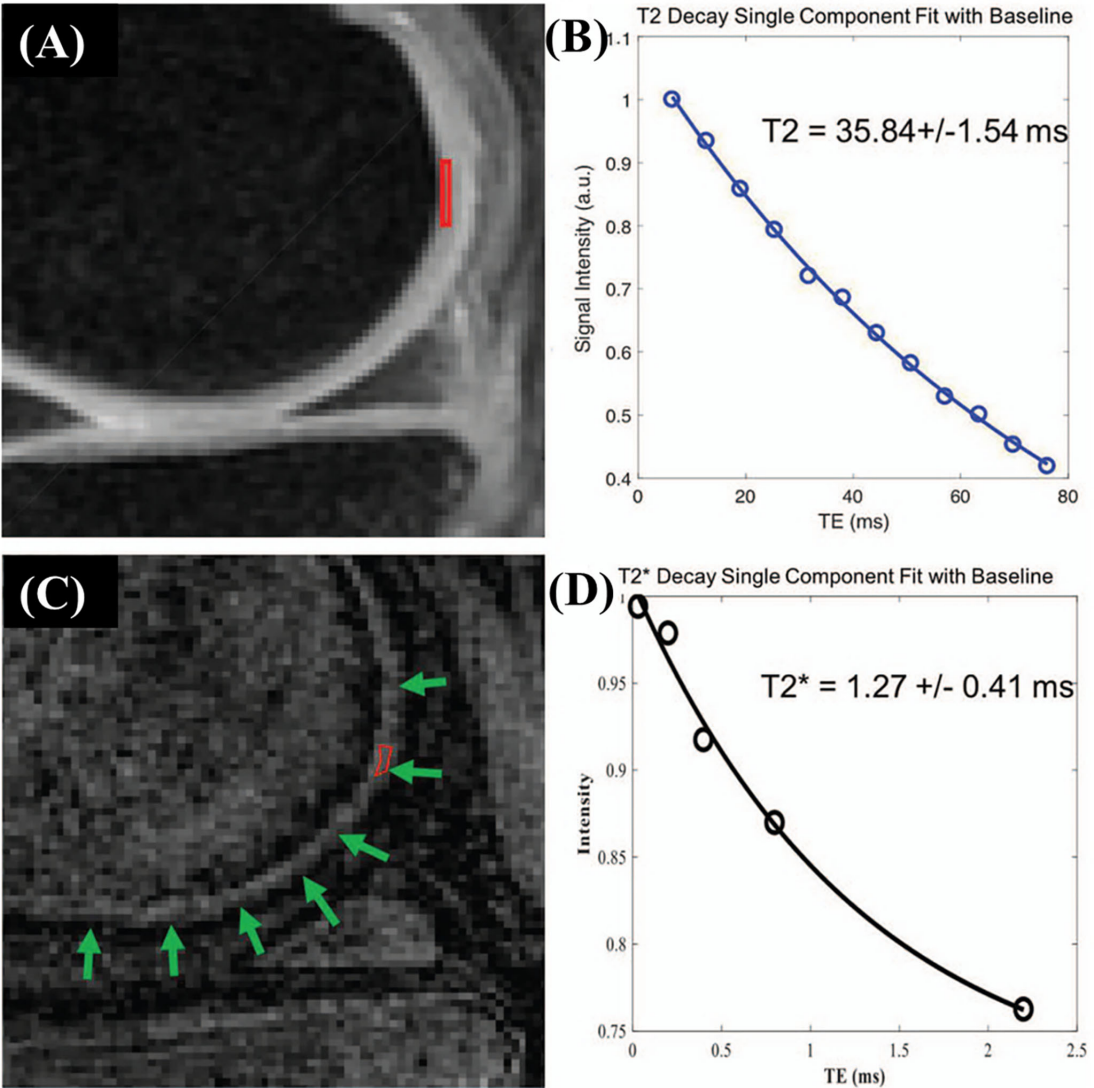
Cadaveric knee joint sample of a 63-year-old female. MR imaging of articular cartilage applying CPMG-T_2_ **(A)** and 3D fat saturated UTE Cones sequences **(C)**. The clinical FSE and CPMG sequences demonstrate signal void for the ZCC region. A single-component exponential fitting curves showed T_2_ values of 35.84 ± 1.54 ms in the deep cartilage **(B)**. The 3D fat-saturated UTE Cones sequence demonstrates high signal but low contrast in the ZCC region (green arrows), with ${\text{T}}_{2}^{*}$ values of 1.27 ± 0.41 ms **(D)**. Modified, with permission from Ref (26).

UTE ${\text{T}}_{2}^{*}$ measurement of cartilage by acquiring UTE images at varying TEs has been widely investigated (42). **Figure 4** shows the comparison between MR imaging of articular cartilage using CPMG-T_2_ (A) and 3D fat-saturated UTE Cones sequences (C) with corresponding single component fitting curves (B) and (D), respectively.

**Figure 5:**
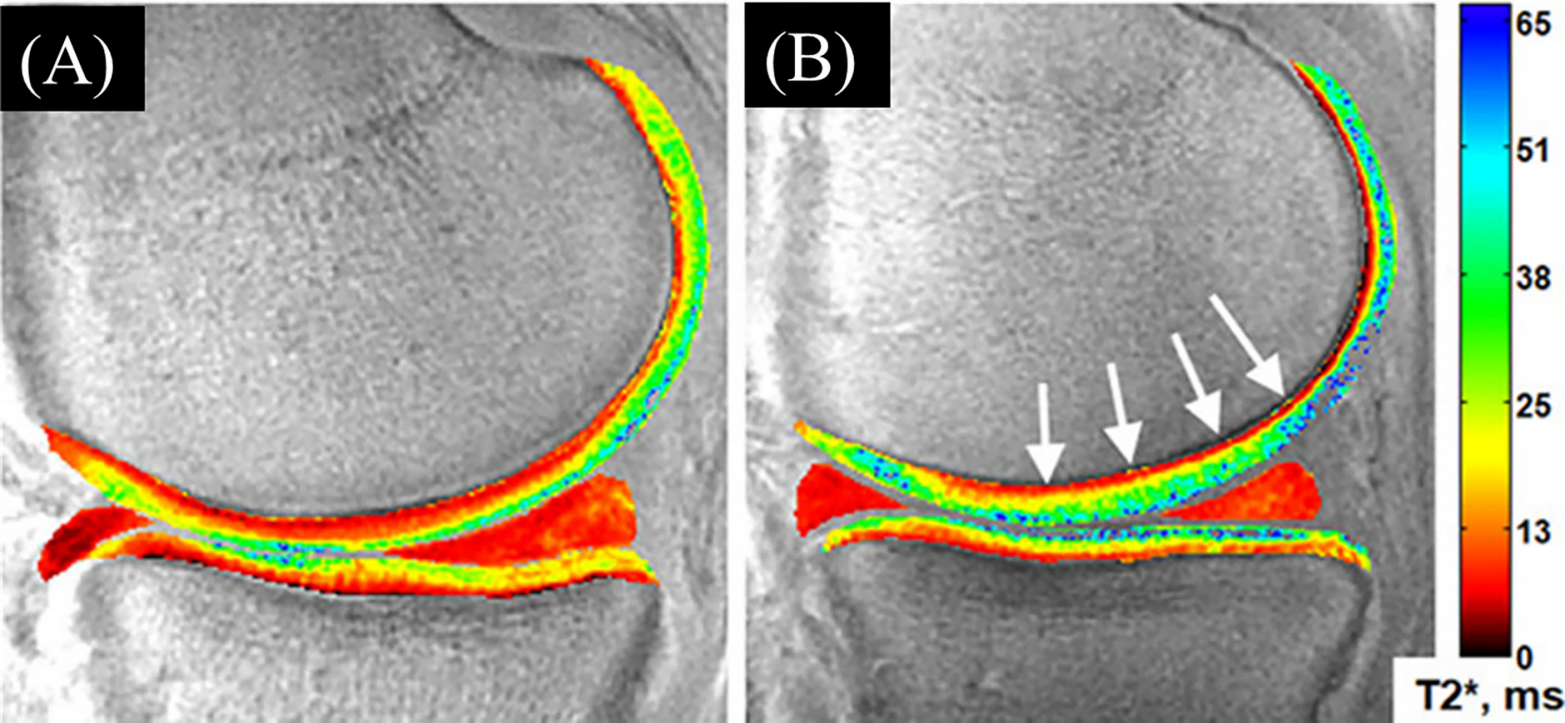
A sample of 2D UTE- ${\text{T}}_{2}^{*}$ maps. **(A)** An uninjured 29-year-old male control subject with typical laminar appearance to UTE- ${\text{T}}_{2}^{*}$ values. **(B)** 34-year-old male ACL reconstruction subject 2 years following surgery, without morphological evidence of medial cartilage (Outerbridge grade 0) pathology, demonstrates elevations to UTE- ${\text{T}}_{2}^{*}$ values throughout the medial femoro-tibial cartilage region, specifically in deep medial femoral cartilage (white arrows). Modified, with permission from Ref (15).

Williams et al. investigated feasibility and repeatability of UTE ${\text{T}}_{2}^{*}$ mapping at 3T *in vivo* and reported coefficients of variation (CVs) lower than 10% (38). Their earlier study also concluded that ${\text{T}}_{2}^{*}$ was not only sensitive to cartilage matrix degenerations but was also able to capture signals from short T_2_ tissues, particularly the deeper regions of severely damaged cartilage that had low ${\text{T}}_{2}^{*}$ values (**Figure 5**) (39).

In several prospective studies performed by Williams et al., UTE ${\text{T}}_{2}^{*}$ mapping has shown potential in cartilage screening following anterior cruciate ligament (ACL) reconstruction (ACLR) in order to predict future osteoarthritis (15, 37, 39). In a two-year study of patients after ACLR, 2D and 3D UTE ${\text{T}}_{2}^{*}$ mappings of the medial femoral cartilage (MFC) and medial tibial plateau (MTP), respectively, were implemented at 3T. Approximately half of the patients demonstrated pre-osteoarthritic changes in the cartilage, indicated by consistent ${\text{T}}_{2}^{*}$ elevations detected in the medial tibio-femoral deep cartilage (15). In another relevant study, Williams et al. reported moderate correlations between UTE ${\text{T}}_{2}^{*}$ depth-wise change rates (profile slopes) with clinical parameters of patient reported outcomes (PROs) and walking mechanics such as knee adduction moment (KAM) in the two years following ACLR (37). In a similar study, Titchenal et al. confirmed that in the two years following ACLR, deep UTE- ${\text{T}}_{2}^{*}$ elevation in medial knee cartilage components was associated with clinical biomarkers of OA, such as increased varus alignment and increased KAM (40). Later, Chu et al. demonstrated the capability of UTE ${\text{T}}_{2}^{*}$ in detecting deep cartilage matrix changes. This study suggested that the return of elevated ${\text{T}}_{2}^{*}$ values in post-ACLR patients to levels comparable with healthy controls might indicate healing (42).

In a pilot study, Foreman et al. incorporated ${\text{T}}_{2}^{*}$ mapping at 3T to evaluate mineralization of deep cartilage layer in a cohort (n=10) of type 2 diabetes mellitus patients as a prototype for vascular insufficiencies. They reported lower ${\text{T}}_{2}^{*}$ values and more mineralized deep cartilage layer in this patient cohort (35). Similarly, Drygalsky et al. investigated UTE ${\text{T}}_{2}^{*}$ mapping as a method to quantify iron concentration caused by internal joint bleeding in the cartilage of hemophilic patients (36). They have reported correlations between joint deterioration and cartilage hemosiderin levels, as detected by decreased ${\text{T}}_{2}^{*}$ .

With the ultimate goal of assessing the biomechanical properties of tissue, there is the question of whether and how ${\text{T}}_{2}^{*}$ and magnetization transfer ratio (MTR) obtained *via* UTE MRI correlate with the mechanical properties of human patellar cartilage. Using an *in situ* model, Hananouchi et al. applied a micro-indentation device with tri-axial force sensor to demonstrate a positive correlation between stiffness/elastic modulus and each predictor variable: UTE- ${\text{T}}_{2}^{*}$ , UTE-MTR, and probing device force (52). In addition, multiple linear regression analyses demonstrated that an even stronger correlation was achieved when all three predictors were combined, further confirming the potential of this noninvasive imaging approach for *in situ* evaluation of the mechanical properties of cartilage tissue.

Evaluation of the zone of calcified cartilage (ZCC) is another important application of ${\text{T}}_{2}^{*}$ mapping in cartilage assessment. Liu et al. used UTE to assess ${\text{T}}_{2}^{*}$ in healthy and cadaveric specimens at 3T and directly visualized and quantified the ZCC with high SNR (26). Advantages of the UTE approach are limited, however, by long scan times and susceptibility to the magic angle effect, both factors that need to be addressed accordingly to maximize the technique’s usefulness.

**Figure 6:**
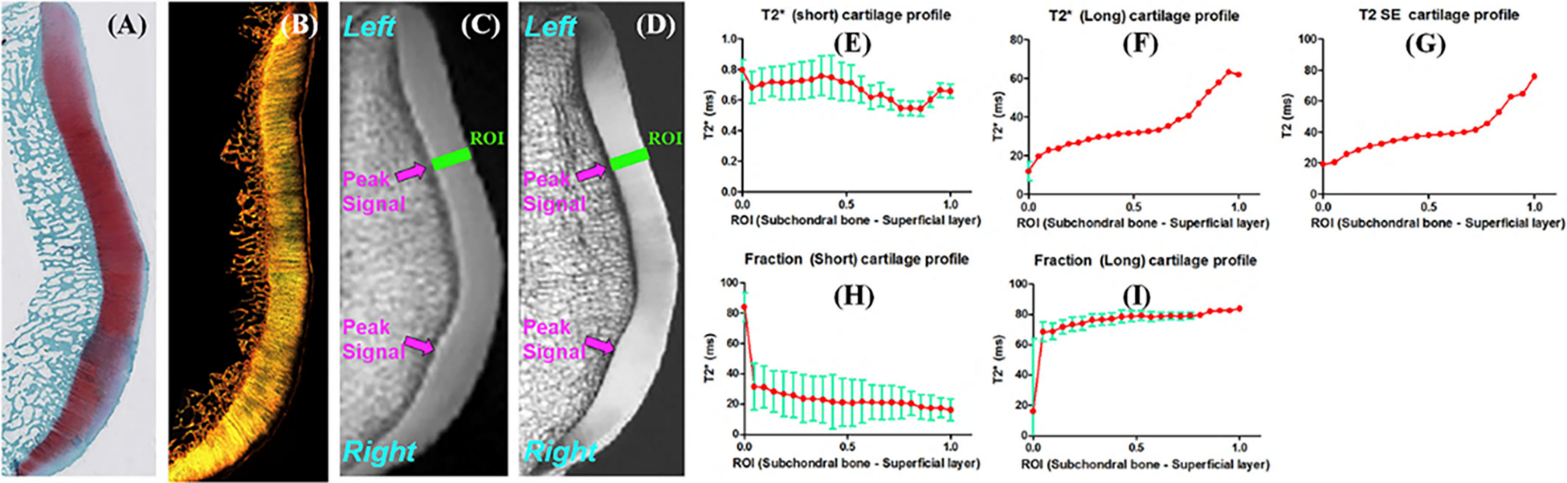
Safranin-O **(A)**, polarized light microscopy (PLM) **(B)**, UTE **(C)**, and PD-SE **(D)** images of a normal sample of patella donated by a 58 year old male donor. Line profiles for short ${\text{T}}_{2}^{*}$ **(E)**, long ${\text{T}}_{2}^{*}$ **(F)**, short fraction **(H)**, and long fraction **(I)**, as well as CPMG T_2_ **(G)** are illustrated. Gradual increases in long ${\text{T}}_{2}^{*}$ , long fraction, and T_2_ from the deep to the superficial cartilage is observed. Fitting errors in single component T_2_ and bi-component ${\text{T}}_{2}^{*}$ analysis are depicted. Peak signal areas corresponding to magic angle on the UTE and SE images are also delineated (arrows). Modified, with permission from Ref (41).

Most of the cartilage UTE ${\text{T}}_{2}^{*}$ studies presented above have employed single component exponential fitting models to calculate the ${\text{T}}_{2}^{*}$ values. Bicomponent UTE ${\text{T}}_{2}^{*}$ analysis has been proposed for more comprehensive cartilage assessment by modeling the tissue as two different components: short ${\text{T}}_{2}^{*}$ and long ${\text{T}}_{2}^{*}$ components (**Figure 6**). Shao et al. identified relatively constant ${\text{T}}_{2}^{*}$ values for short ${\text{T}}_{2}^{*}$ components across various cartilage depths, but noted increasing ${\text{T}}_{2}^{*}$ values for the long ${\text{T}}_{2}^{*}$ components (41). The fraction of the long ${\text{T}}_{2}^{*}$ component was found to increase from the calcified toward the superficial cartilage.

To determine the correlation between short and long ${\text{T}}_{2}^{*}$ water fractions obtained by UTE MRI with histopathologic and polarized light microscopic (PLM) results, Pauli et al. scanned human cadaveric patellar cartilage by applying UTE-MRI, spin-echo imaging, and subsequent bicomponent analysis. They realized that short ${\text{T}}_{2}^{*}$ had significant correlations with both the Mankin (histopathologic) scores and Vaudey (PLM) scores (54), suggesting that short ${\text{T}}_{2}^{*}$ could potentially serve as a biomarker of cartilage degeneration. In another study, Du et al. applied 2D UTE with bicomponent analysis on a 3T scanner to quantify short and long ${\text{T}}_{2}^{*}$ as well as their fractions in cadaveric patellar cartilage (0.48 ms and 34.97 ms; 18.47% and 81.53%, respectively) (55). Jang et al. proposed another variant of the technique: a single scan ramped hybrid encoding (RHE) sequence at 3T to provide rapid bicomponent ${\text{T}}_{2}^{*}$ analysis of the human knee joint with a total scan time of less than 9 minutes (56).

Multicomponent ${\text{T}}_{2}^{*}$ UTE acquisitions have also proved to be feasible in knee cartilage studies (53). This approach is based on two important fundamental observations in the literature: 1) that disorganization of collagen fibers is a sign of early stage cartilage degeneration, and 2) that water molecules trapped within well-organized collagen fibrils are sensitive to collagen alterations (53). Based on these facts, Qian et al. developed a UTE sequence and applied it to *ex vivo* human tibial plateau cartilage specimens in a 3T scanner under the minimum TE of 0.5 ms (53). They demonstrated that multicomponent ${\text{T}}_{2}^{*}$ UTE mappings could probe the short T_2_ relaxations of the water molecules trapped in the collagen matrix. Most cartilage pixels were found to have mono- and/or bicomponent ${\text{T}}_{2}^{*}$ relaxations with short ${\text{T}}_{2}^{*}$ values of 1-6 ms and long ${\text{T}}_{2}^{*}$ values of about 22 ms across a wide range of component intensity (0-100%). The multi-component ${\text{T}}_{2}^{*}$ decay map has effectively indicated the decay types (e.g., mono-, bi-, tri-, and nonexponential) for each pixel in cartilage.

### UTE T_1_

**Figure 7:**
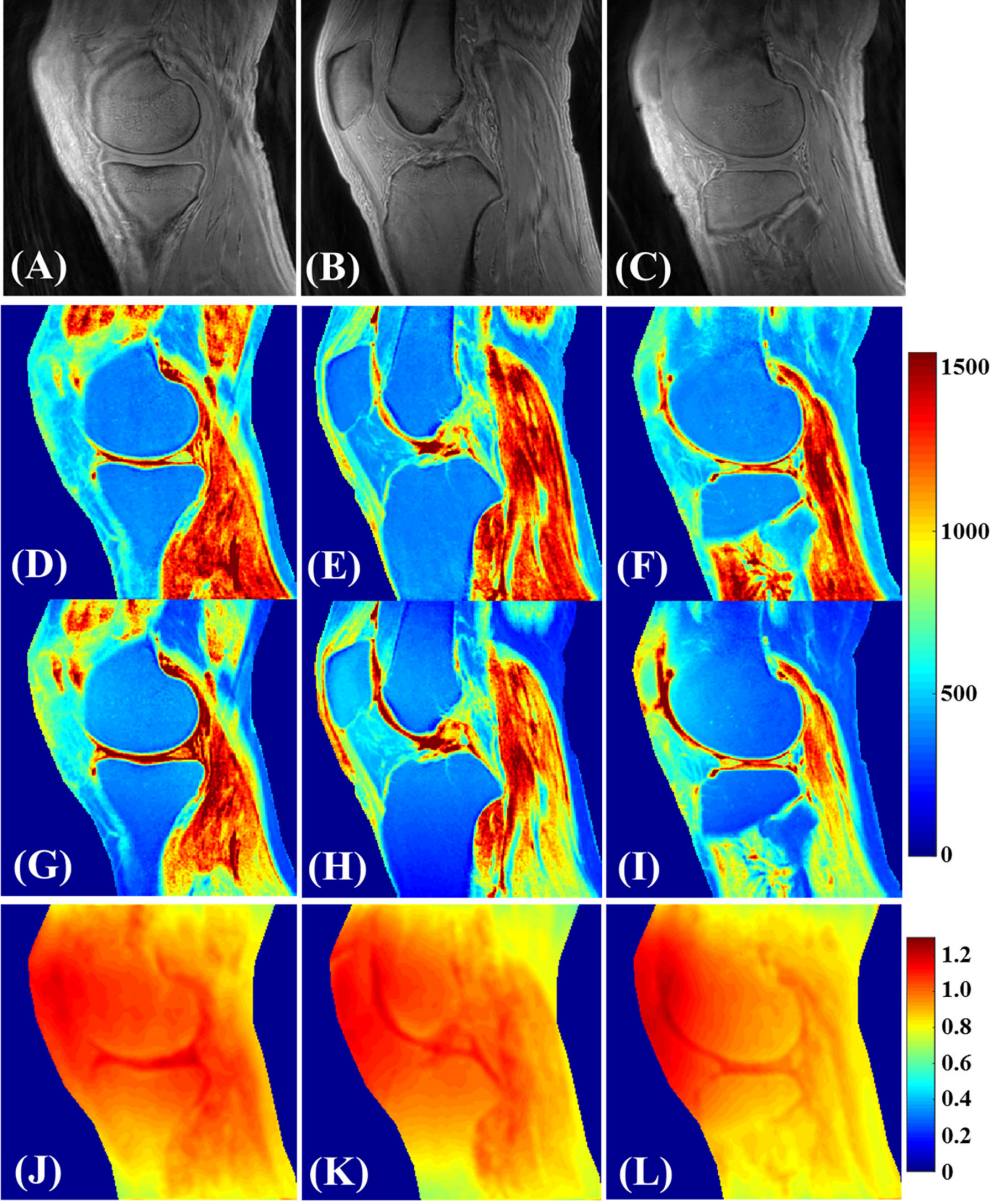
Knee tissues in a healthy 35-year-old male volunteer **(A–L)**. **(A–C)** are the selected VFA images with FA=5°. T_1_ mapping utilizing both the proposed 3D UTE-Cones AFI-VFA **(D–F)** and B1-uncorrected VFA **(G–I)** methods are illustrated. The B_1_ maps generated by the AFI technique **(J–L)** are also depicted. T_1_ estimation errors as a result of B_1_ inhomogeneity in the images of **(G–I)** have been corrected by the proposed 3D UTE-Cones AFI-VFA technique, purticularly in areas close to the coil boundary. Modified, with permission from Ref (31).

**Figure 8:**
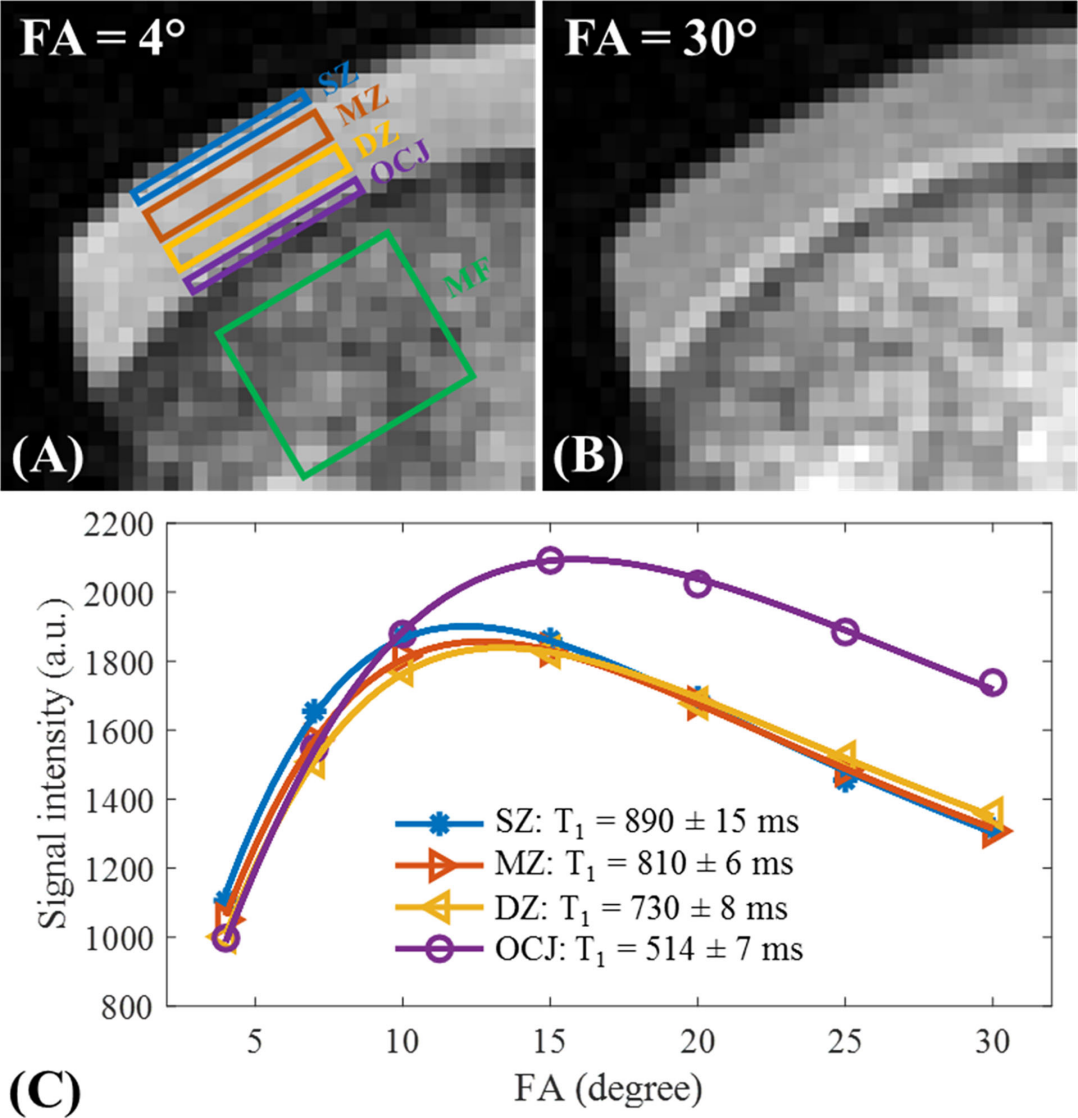
T_1_ measurements of a sample of patellar cartilage in the superficial zone (SZ), middle zone (MZ), deep zone (DZ), and OCJ region. The bone marrow fat (MF) section has also been labeled in **(A)** Images with respective flip angles of 4° and 30° are demonstrated in **(A, B)** In the image with the flip angle of 30°, a high signal intensity band can be seen in the OCJ region. Image **(C)** demonstrates the fitting curves and T1 values for the SZ, MZ, DZ, and OCJ. Gradual decrease of T1 values from SZ to OCJ is observed. Modified, with permission from Ref (28).

Cartilage T_1_ measurement using UTE images can be performed by acquiring images with varying TRs or FAs (31). However, achieving an accurate FA for tissues with short ${\text{T}}_{2}^{*}$ values is challenging due to inhomogeneities in the B1 field. In a feasibility study for T_1_ values in different knee joint tissues of healthy young volunteers in a 3T scanner, Ma et al. used a single exponential fitting model over different FAs by considering the actual FAs corrected by achievable B1 value over the cartilage volume (31). This so-called UTE actual flip angle-variable flip angle (UTE-AFI-VFA) method was able to measure the T_1_ value with B_1_ correction for all the major tissues in the knee with T_2_s greater than 1 ms, which included approximately all of the articular cartilage regions (**Figure 7**). A similar UTE method was later used by Cai et al. to measure the T_1_ values of human patellar cartilage layers; they reported significantly shorter T_1_ values for the deepest layer compared with other layers (28). **Figure 8** demonstrates T_1_ measurements for different layers of a patellar cartilage sample including the superficial zone (SZ), middle zone (MZ), deep zone (DZ), and osteochondral junction (OCJ) region.

The UTE T_1_ measurement method described above has been utilized as an important input for UTE magnetization transfer (MT) modeling (34).

## Inversion Recovery UTE (IR-UTE)

**Figure 9:**
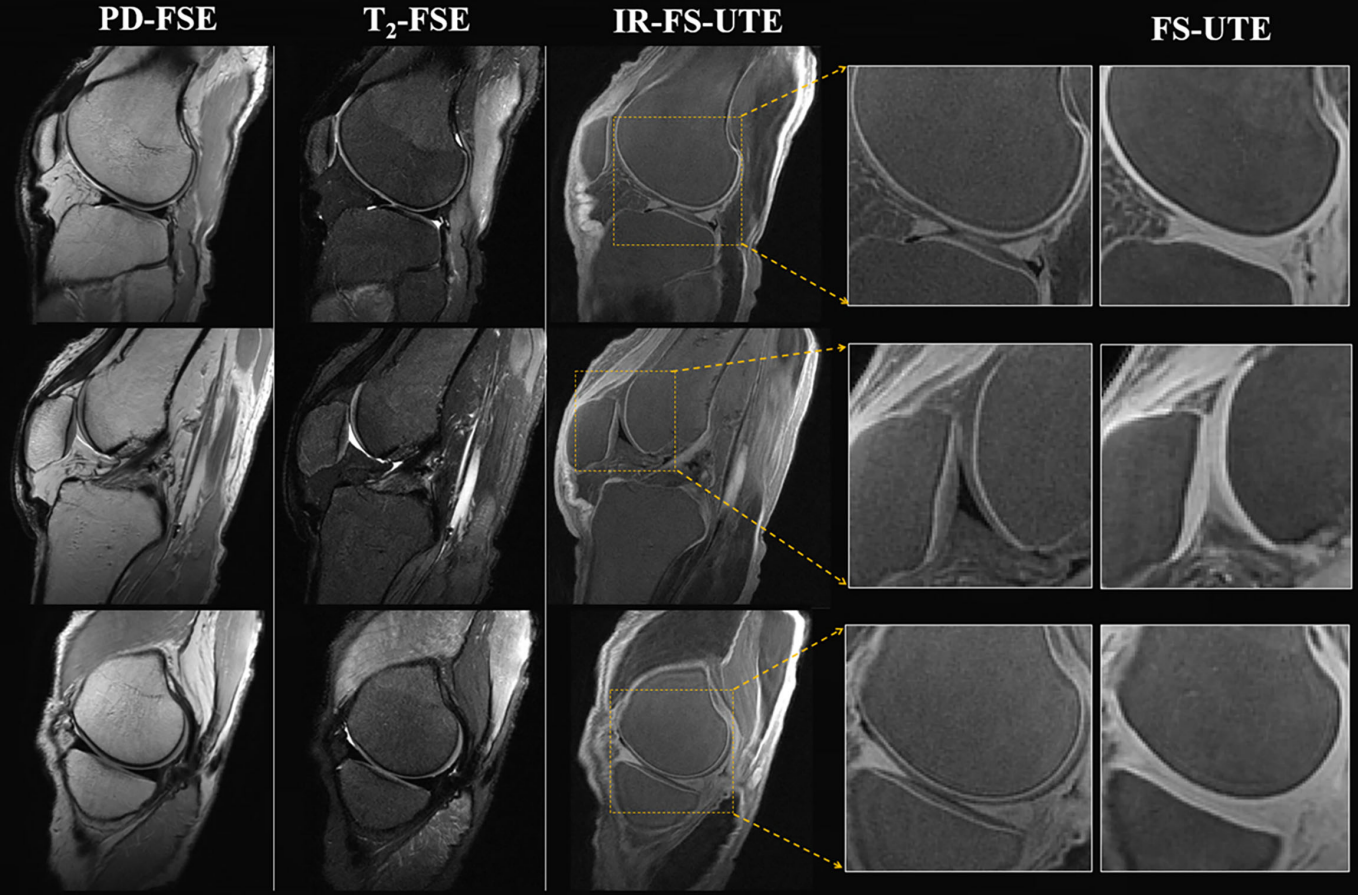
Image of the OCJ region in a normal *ex vivo* knee joint sample from a 31-year-old male donor. The clinical images (PD-weighted FSE in first column and T_2_-weighted FSE in second column) are employed to compare with the T_1_-weighted IR-UTE-Cones images (third column). High OCJ contrasts (i.e., bright band) are demonstrated in the IR-UTE-Cones images, which are more visible in the zoomed images. The last column consists of the conventional fat-saturated UTE-Cones images for the purpose of comparison. These demonstrate signal from both calcified and uncalcified cartilage. Modified, with permission from Ref (12).

UTE MRI has been shown to be capable of visualizing the superficial and deep layers of articular cartilage. Deep layer cartilage and ZCC zones are of great interest to researchers because of their roles in the progression of OA; consequently, several inversion recovery (IR) preparation pulses have been proposed to be used in combination with UTE MRI for visualization and quantification of these clinically significant cartilage layers (12, 68). **Figure 9** compares clinical images (PD‐weighted FSE in first column and T_2_‐weighted FSE in second column) with UTE images (IR prepared fat saturated UTE (IR-FS-UTE) in the third and fourth columns and FS-UTE in last column).

In most IR-UTE techniques, an adiabatic full passage inversion pulse is used before the UTE acquisition to invert the longitudinal magnetization of water without incurring a B1 inhomogeneity penalty. Pure short T_2_ water pool imaging depends on the optimized inversion time (TI) which is required to let the inverted long T2 magnetization reach the nulling status. UTE MRI acquisition after the nulling point results in short T_2_ water visualization, which is dominant in the deep layer and ZCC of articular cartilage. Maximizing the short T_2_ signal while simultaneously avoiding long T_2_ signal contamination has been investigated by Ma et al. and Jang et al. (12, 68) who have optimized several sequence parameters such as TI.

**Figure 10:**
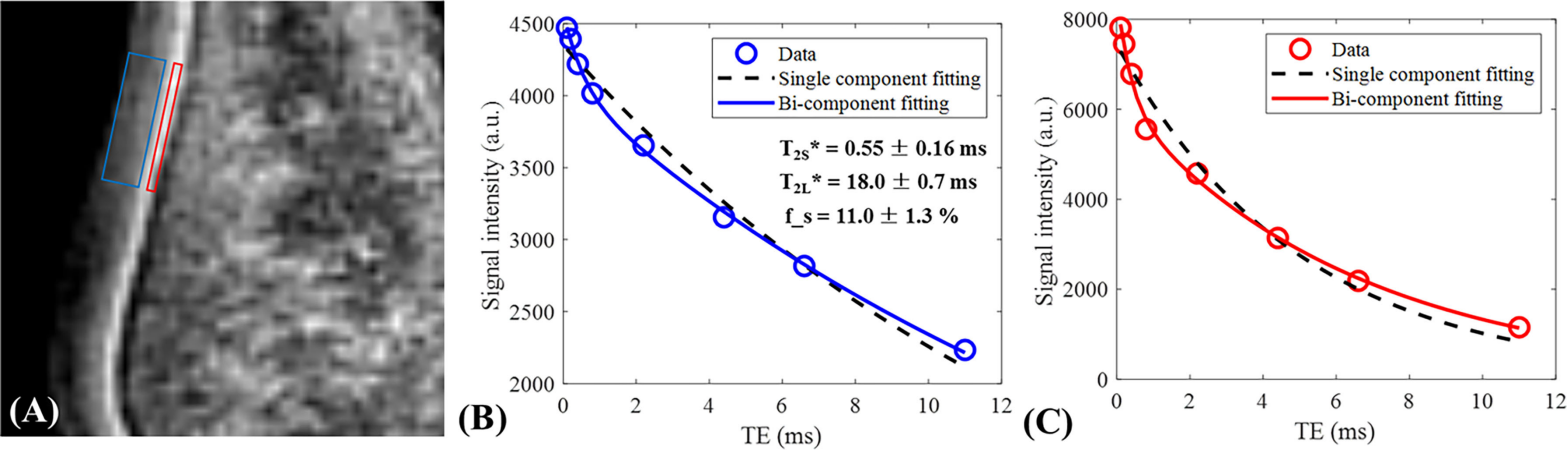
Single- and bi-component analysis of IR-FS-UTE imaging of more superficial cartilage (blue region) and OCJ (red region in **A**) regions. The bi-component model performs much better than the single-component model in data fitting **(B, C)**. For the more superficial cartilage region, the ${\text{T}}_{2}^{*}$ values of short and long T_2_ components as well as the short T_2_ fraction were 0.55 ms, 18.0 ms, and 11.0%, respectively **(B)**. For the deeper cartilage region, the ${\text{T}}_{2}^{*}$ values of the short and long T_2_ components as well as the short T_2_ fraction were 0.38 ms, 6.5 ms, and 27.0%, respectively **(C)**.

**Figure 10** shows the single- and bi-component analysis of IR-FS-UTE imaging of more superficial cartilage (blue box) and OCJ (red box) regions. As can be seen from these fitting curves, the bi-component model performs much better than the single-component model with regard to data fitting. The ${\text{T}}_{2}^{*}$ relaxations for both the short and long T_2_ components in the more superficial cartilage region are longer than those in the OCJ region. Higher short T_2_ fraction is also found in the OCJ region. These results demonstrate that the collagen matrix is more densely distributed or there is more calcification existed in the OCJ region.

For deep cartilage and ZCC cartilage layer assessment, dual adiabatic IR-UTE (Dual-IR-UTE) has also been developed as a method that avoids fat signal contamination that may not be nulled by just a single adiabatic IR pulse (13, 14). Dual-IR-UTE is designed to employ two adiabatic IR pulses that invert and suppress long T_2_ water and fat magnetizations at their respective frequencies.

By utilizing Dual-IR-UTE images at differing TEs, Dual-IR-UTE images with differing TRs, and spin locking-prepared Dual-IR-UTE acquisitions, the ZCC were able to be visualized and quantified (i.e., ${\text{T}}_{2}^{*}$ , T_1_, and T_1ρ_) (13).

## Off-Resonance Saturation (ORS) and Magnetization Transfer (MT) UTE

The application of off-resonance saturation (ORS) pulses has been suggested by Du et al. (69) and Carl et al. (70) as a way to optimize the UTE MRI contrast of short T_2_ tissues such as deep layer cartilage. Because the ORS pulse application selectively reduces the short T_2_ signals of the surrounding tissues and fluids, subtracting the UTE images with and without applying ORS facilitated selective positive short T_2_ enhancement, which was a result of the ORS saturation. These studies demonstrated that ORS UTE could efficiently suppress long T_2_ while highlighting short T_2_ signals. It should be noted that the contrast can be improved by increasing the ORS pulse power while decreasing the ORS frequency difference from the water peak.

Using higher power levels, the saturation induced by the ORS pulse can be applied to proton pools with extremely short T_2_ values, as is the case in macromolecules such as collagen. The saturation induced in the macromolecular proton pool, for instance, would then be transferred to the surrounding water proton pools, including bound and free water pools. The magnetization transfer phenomenon can be employed in UTE-MT imaging for macromolecular pool quantifications with respect to the water pool. UTE-MT ratio (UTE-MTR) is defined as (unsaturated - saturated)/unsaturated. UTE-MTR values have been compared to ${\text{T}}_{2}^{*}$ and T_2_ mapping values in a study by Yang et al. on 20 degenerative anterolateral condyles of total knee arthroplasty specimens on a 3T scanner (19). This study observed strong correlations between Mankin histological scores (which were used as an indication of the cartilage degeneration level) with the resultant UTE-MTR.

In an *ex vivo* study, Shao et al. investigated the diagnostic efficacy of multiparametric quantitative UTE MRI for knee cartilage degeneration detection (71). They obtained 20 anterolateral femoral condyle specimens from total knee arthroplasty patients and scanned them on a 3T scanner using UTE-MT, UTE-AdiabT_1ρ_, UTE- ${\text{T}}_{2}^{*}$ , and CubeQuant-T_2_ sequences. They also classified cartilage degeneration according to polarized light microscopy (PLM) collagen organization score and OA Research Society International grade. The study demonstrated the strongest correlation amongst all the investigated biomarkers between UTE-MTR and both abovementioned cartilage degeneration classifications. The receiver operating characteristic (ROC) analysis revealed that UTE-MTR also possessed a higher diagnostic efficacy for mild cartilage degeneration than the other biomarkers.

As previously mentioned, Hananouchi et al. (52) demonstrated a positive correlation between the mechanical properties of human patellar cartilage such as stiffness/elastic modulus and MR properties such as UTE-MTR by using an indentation device.

**Figure 11:**
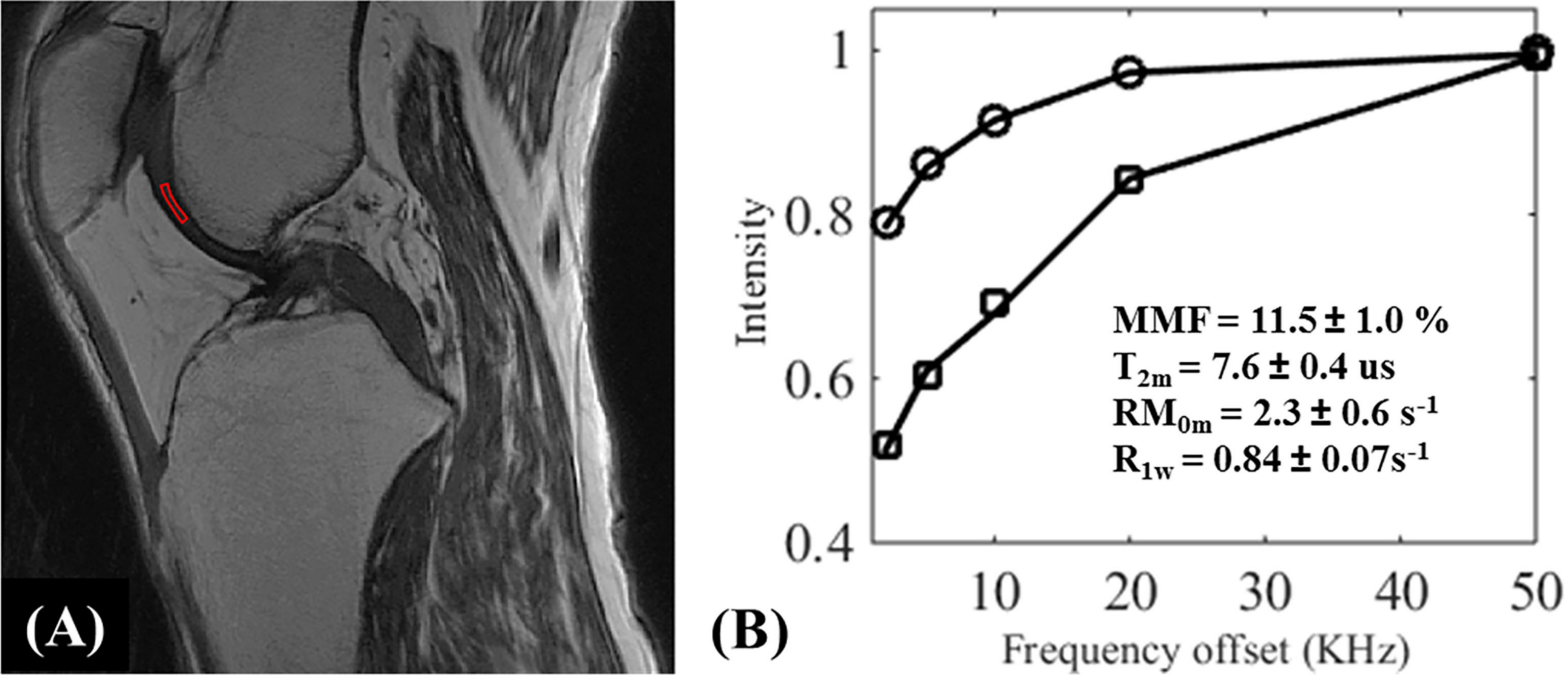
UTE-MT modeling (fitting) of knee cartilage. The UTE‐Cones‐MT **(A)** images were acquired from an *ex vivo* knee joint specimen with two different MT flip angles of 500° and 1500° at five different frequency offsets of 2, 5, 10, 20, and 50 KHz, and a region of interest (ROI) in the femoral condyle cartilage was selected for MT modeling. Image **(B)** illustrates the fitting curve and corresponding fitted parameters [i.e., MMF (%), T_2m_ (us), RM_0m_ (s^-1^), and R_1w_ (s^-1^)]. MMF = macromolecular fraction; T_2m_ = T_2_ relaxation time of macromolecular pool; R_1w_ = spin-lattice relaxation rate of water pool; RM_0m_ = proton exchange rate from water to macromolecular pool.

**Figure 12:**
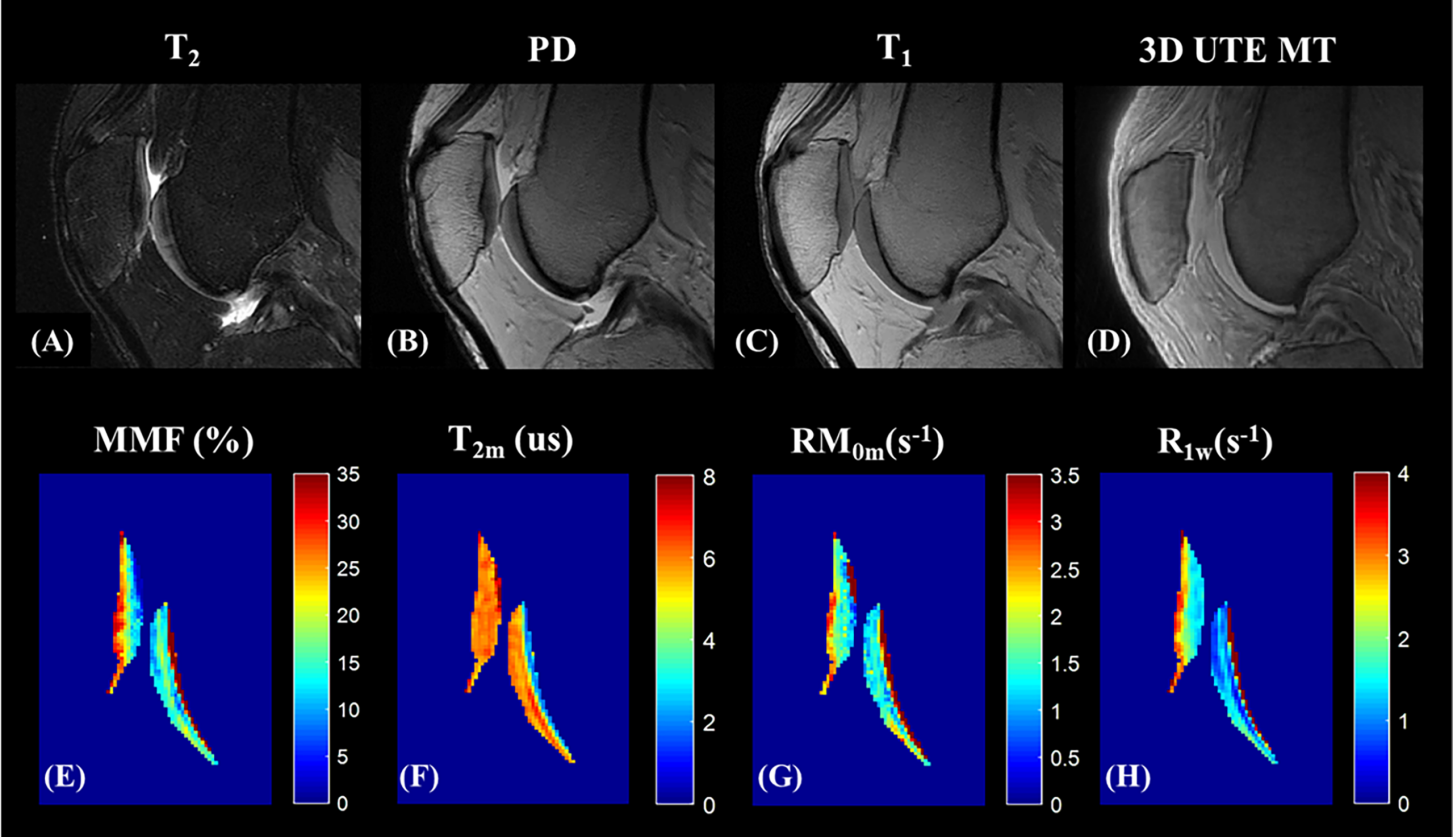
UTE-MT modeling (mapping) of knee cartilage. Panels **(A–D)** show the representative *ex vivo* knee MR images acquired from clinical sequences **(A–C)** and 3D UTE -MT (MT flip angle of 500° and frequency offset of 50 KHz) **(D)**. Color mapping of UTE-MT modeling parameters, including MMF (%) **(E)**, T_2_ relaxation time of the macromolecular pool (T_2m_, us) **(F)**, proton exchange rate from water to macromolecular pool (RM_0m_, s^-1^) **(G)**, and spin-lattice relaxation rate of the water pool (R_1w_,s^-1^) **(H)** are demonstrated.

Because MTR values are functions of the MT pulse power level and the frequency offset, MTR is difficult to reproduce between studies. UTE-MT modeling has therefore been proposed to provide multiple parameters, including macromolecular fraction (MMF), macromolecular relaxation time (T_2_mm), and exchange rates, while preserving much higher levels of reproducibility (18, 72). **Figures 11**, **12** show the MT modeling fitting curve and parameter maps, respectively, of cartilage from a representative knee joint sample. Recent studies also demonstrate that UTE-MT modeling is nearly insensitive to the magic angle effect (73, 74), supporting its potential for effective detection of cartilage degeneration.

Jerban et al. assessed UTE-MT variation under mechanical loading in the tibiofemoral cartilage of cadaveric knee joints and calculated MMF from UTE-MT modeling (58). To assess MRI data differences between loading conditions, they applied Wilcoxon rank sum test. For young specimens and with load increases, MMF increased in all grouped regions of interest (ROIs) of the tibial articular cartilage, femoral articular cartilage, and articular cartilage regions that were both covered and uncovered by meniscus. MMF increases were significant for articular cartilage regions covered by meniscus. After unloading, MMF decreased in all studied regions, but only reaching significant levels of difference for the articular cartilage regions covered by meniscus. For elderly specimens, they did not observe significant changes in MRI parameters by loading or unloading. This study of different patterns of MMF variations in the joints of young and elderly samples demonstrates the capability of UTE-MT modeling combined with knee loading in differentiating between normal and abnormal knee joints.

In a prospective study, Xue et al. investigated the feasibility of MMF to differentiate normal and degenerated knee cartilage (20). They employed a 3D UTE-MT sequence on 62 volunteers with and without osteoarthritis at 3T. A two-pool MT model was applied to evaluate the MMF difference between cartilage in normal and abnormal knees as categorized by both Kellgren-Lawrence (KL) grades and Whole-Organ Magnetic Resonance Imaging Scores (WORMS). Reporting significant negative correlations of MMF with KL grade and WORMS, this study is yet another indication of the MMF potential to detect early OA.

Namiranian et al. investigated the correlations of MTR, MMF, and T_2_mm with the mechanical properties of articular cartilage, namely cartilage stiffness and Hayes elastic modulus, scanned on a 3T scanner (34). Correlations were assessed in the superficial layer, deep layer, and global (combining both the superficial and deep layers) cartilage ROIs. Higher correlations of mechanical properties were found with MMF and MTR at the superficial layer compared with either the deep layer or the global ROI, likely because the indentation tests measure the surface mechanical properties.

## UTE-T_1ρ_

T_1ρ_ relaxation occurs after the application of a long-duration on-resonance RF pulse in order to “spin-lock” the magnetization vector into a rotated frame (75). T_1ρ_ relaxation time is always higher than T_2_ relaxation time. The T_1ρ_ biomarker has been hypothesized to be sensitive to slow-motion interactions between protons of constrained water molecules and those of associated macromolecules in the extracellular matrix of musculoskeletal tissues, such as proteoglycans (PG) in articular cartilage (75). The conventional T1ρ cannot be used for all cartilage zones, particularly the ZCC, due to the lack of signal in tissue with short T_2_ values. However, UTE-T_1ρ_ techniques, either with continuous wave (CW) spin-locking (CW-UTE-T_1ρ_) or with adiabatic spin-locking (UTE-AdiabT_1ρ_), have been developed to quantify the T_1ρ_ of short and long T_2_ tissues (17).

**Figure 13:**
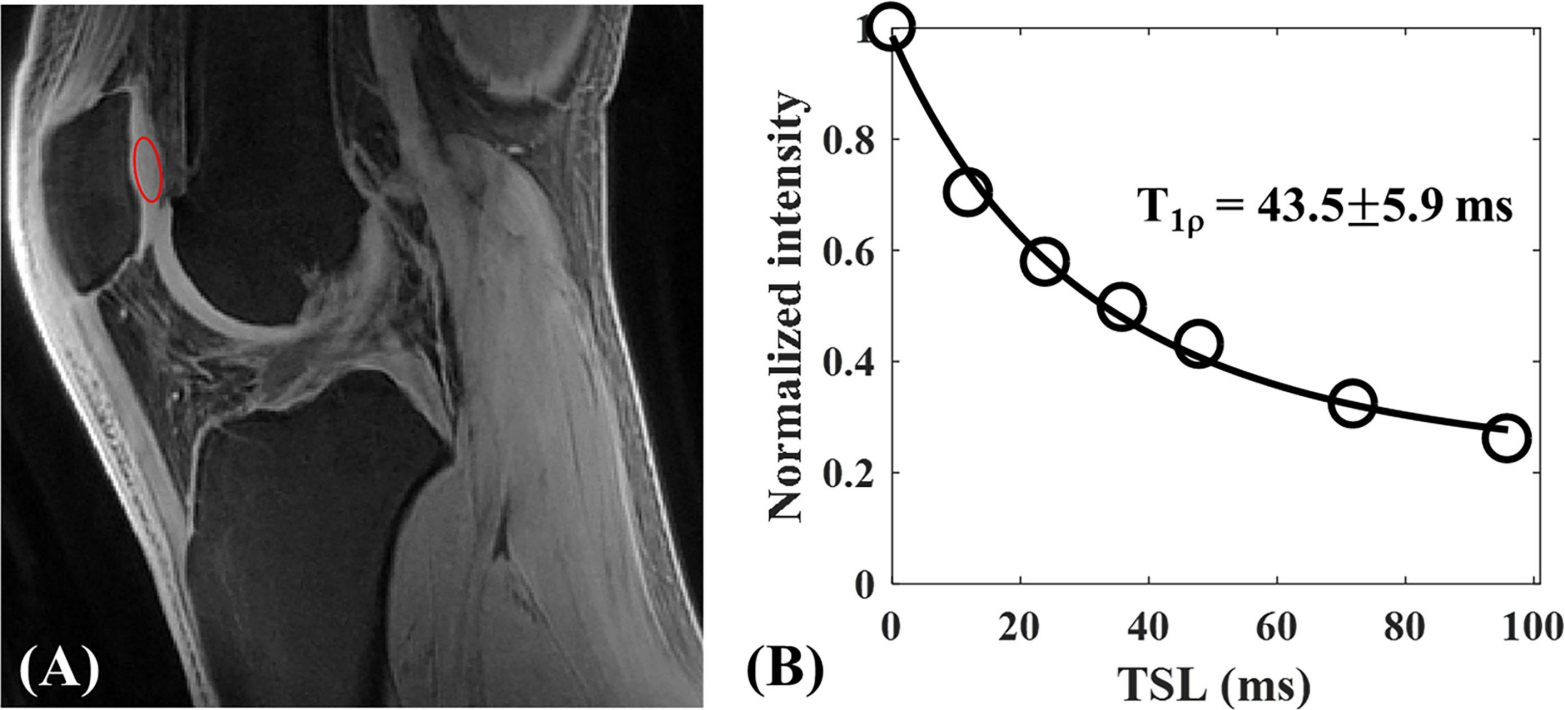
3D UTE-AdiabT_1ρ_ Cones imaging of an *in vivo* knee from a 23-year-old healthy male volunteer. Representative AdiabT_1ρ_ image **(A)** with region of interest (ROI) (red circle) and corresponding fitting curve **(B)** of patellar cartilage are demonstrated. The T_1ρ_ value of patellar cartilage was obtained with 43.5 ± 5.9 ms. Modified, with permission from Ref (17).

**Figure 14:**
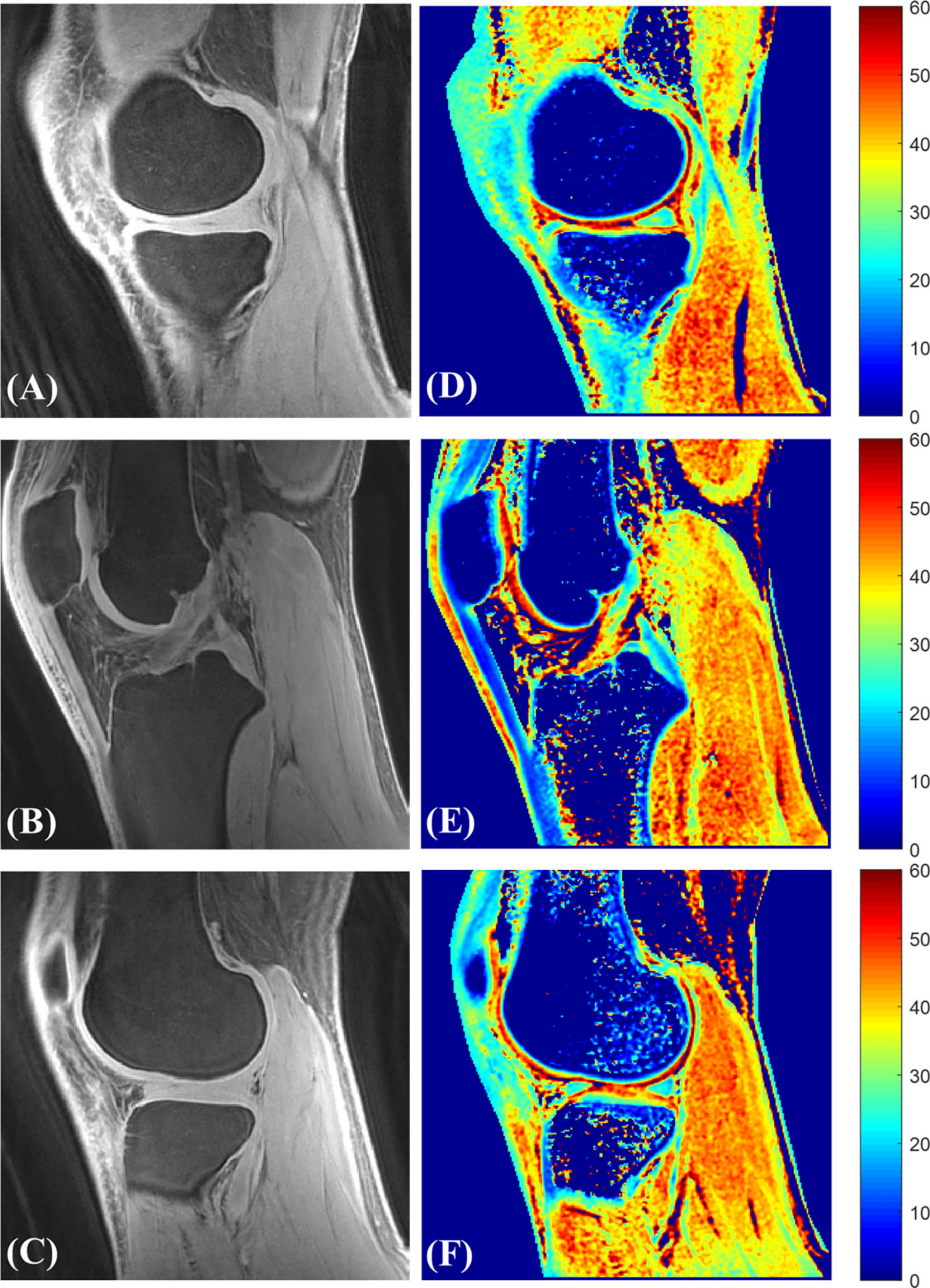
Representative 3D UTE-AdiabT_1ρ_ Cones images **(A–C)** with their corresponding T_1ρ_ maps **(D–F)**.

Ma et al. has developed a novel UTE-AdiabT_1ρ_ sequence that uses an adiabatic spin-lock pulse train followed by UTE data acquisition (17). They have achieved robust *in vivo* and *ex vivo* volumetric measurements of T_1ρ_ in both short and long T_2_ tissues of the knee joint, including patellar cartilage. **Figures 13**, **14** show the representative UTE-AdiabT_1ρ_ fitting curve and parameter maps, respectively, for *in vivo* knee joint cartilage.

A recent study by the same research group demonstrated that this UTE-AdiabT_1ρ_ sequence had a low sensitivity to the magic angle effect (57). Wu et al. scanned human patellar samples at 3T and investigated the magic angle effect at five angular orientations ranging from 0° to 90° in relation to the B_0_ field. For the comparison, they also applied UTE‐ ${\text{T}}_{2}^{*}$ and UTE continuous wave T_1ρ_ (UTE‐CW‐T_1ρ_) sequences, ultimately concluding that the 3D UTE-AdiabT_1ρ_ sequence was less sensitive to the magic angle effect across all patellar cartilage layers compared with either the UTE‐CW‐T_1ρ_ or UTE‐ ${\text{T}}_{2}^{*}$ sequences (57).

Namiranian et. al., also reported significant correlations between UTE-AdiabT_1ρ_ and the articular cartilage mechanical properties (34). Higher correlations were found at superficial layer as the indentation tests mainly measured the surface mechanical properties.

Lastly, in an *ex vivo* study by Shao et al. (71), the UTE-AdiabT_1ρ_ values showed significant differences but lower diagnostic efficacy (compared to UTE-MTR) between the normal group and mildly degenerated group of anterolateral femoral condyle specimens obtained from total knee arthroplasty.

## Chemical Exchange Saturation Transfer (CEST) UTE

The chemical exchange saturation transfer (CEST) phenomena occurs when water-soluble macromolecules with exchangeable protons (generally, the amide side of the chains in contrast agents typically injected into joints) become exposed to water or body fluid protons (76). Because CEST is assumed to occur more intensely in an acidic environment where there are more free protons available, it has been hypothesized that quantification of CEST can be used to detect pH changes in tissues, which themselves may be a sign of lactic acid accumulation and pain triggers.

AcidoCEST-UTE has been introduced recently by Ma et al. as a potential technique for measuring extracellular PH in tissues with short T_2_ values such as articular cartilage (50). The feasibility of the acidoCEST-UTE technique was investigated on a liquid phantom and *ex vivo* human cartilage phantoms doped with iodinated contrast agent in a 3T scanner. Among iodinated contrasts tested in liquid phantoms, iopamidol and iohexol were determined to be feasible for pH detection using AcidoCEST-UTE., with iohexol having the best performance in determine the tissue pH (50).

In a similar study, High et al. hypothesized that changes in extracellular pH may mediate the degeneration of cartilage (60). To determine the feasibility of the acidoCEST MRI method for measuring pH in cartilage *in vivo* and to optimize saturation powers used with the technique, they evaluated MTR asymmetry and ratio of RF power mismatch at different powers in cartilage tissue phantoms for iodinated contrasts of iohexol and iopamidol (77). They also administered the iodinated contrasts directly into the joints of 4 patients with chronic knee pain and, by using optimized RF powers, they utilized the acidoCEST-UTE MRI sequence to evaluate the pH of joint tissues and fluid. In the phantoms, the ratio of powers demonstrated the strongest correlation with pH. *In vivo* measurements of acidoCEST-UTE pH of intra-articular fluid were similar to electrode measurements of the contrast media (7.65 vs. 7.5 for iohexol and 7.22 vs. 7.1 for iopamidol, respectively). This study demonstrated that after direct intra-articular administration of either iohexol or iopamidol, acidoCEST-UTE MRI is capable of measuring cartilage pH *in vivo*.

## UTE Spectroscopic Imaging (UTESI)

UTE spectroscopic imaging (UTESI) has been developed by Du et al. (46) as a suite of chemical shift imaging techniques suitable for MSK tissues with short T_2_ values such as articular cartilage particularly at the deep and calcified layers. In general, chemical shift imaging techniques provide spectroscopic information, an estimation of the relative numbers of the protons with a specific relaxation time, in the targeted tissue. UTESI employs a combination of highly under-sampled interleaved projection reconstruction with a multi-echo UTE acquisition at progressively increasing TEs up to few milliseconds in order to investigate the various proton pools in cartilage including the short T_2_ proton pool. The undersampled data is used for ${\text{T}}_{2}^{*}$ calculation through either exponential fitting of the time domain images or line fitting of the magnitude spectrum.

## UTE With Fat Suppression

Fat saturation techniques are regularly used to improve image contrast for better visualization of the target tissue. In a novel study, Ma et al. proposed a soft-hard composite RF pulse to suppress fat signals in UTE imaging for tissues with short T_2_ (78). The soft pulse of this composite pulse has a narrow bandwidth, small negative flip angle, and is centered on the fat peak. The hard pulse is a short rectangular pulse and has a small positive flip angle. The outcome is fat magnetization that tips down and back with an identical flip angle, i.e., that returns to a state of equilibrium, so that only the excited water magnetization remains. Ma et al.’s feasibility study investigated the knees and tibias of respectively five and six healthy volunteers between the ages of 22 and 35 on a 3T scanner. For comparison, a conventional fat saturation (FatSat) model was used; for evaluation of the novel technique’s efficiency, signal suppression ratio (SSR) was introduced. Higher SSR was consistent with better fat suppression or water attenuation induced by fat suppression. The soft-hard composite pulse approach resulted in much lower signal attenuation of water imaging than the conventional FatSat method on simulation studies, performed well for *in vivo* fat suppression, and produced better preservation of both long and short T_2_ signals, significantly higher SSR specifically for short T_2_ signals, and better contrast between water and fat.

Jang et al. developed the ultrashort echo time cones double echo steady state (UTE-Cones-DESS) sequence for fast volumetric and high quality image contrast of MSK tissues (22). This technique was performed in the knee joints of both healthy volunteers and patients with osteoarthritis. To achieve a scan time of less than 5 minutes without compromising image quality, Jang et al. applied fat suppression using a novel, single-point Dixon (spDixon) approach (79). This high contrast morphological imaging method of short T_2_ tissues, including the deep layers of cartilage, has the potential to assess musculoskeletal diseases.

Chen et al. investigated the effects of fat saturation on three quantitative UTE images including ${\text{T}}_{2}^{*}$ , T_1ρ_, and MT (59). These sequences were implemented with and without fat saturation on knee cartilage samples and whole cadaveric knee specimens at 3T. This investigation demonstrated strong correlations and agreement with some minor differences for all UTE biomarkers between the measurements with and without FatSat, confirming that fat suppression was effective in all three UTE sequences where FatSat was employed for whole joint quantification including cartilage.

Springer et al. aimed to visualize short T_2_ tissues with optimal contrast and adequate suppression of signal from the surrounding fat by implementing a time-efficient 1:1 double pulse water-selective excitation (WE) into a 3D UTE sequence (WE-UTE) (80). In this study, they applied WE-UTE on the flexor tendons of the human hand and the PCLs of the knee *in vivo* at 3T and saw that these short T_2_ tissues could be well demarcated with positive contrast compared to surrounding fat. Even for tissues with T_2_  values of 1 ms, WE-UTE led to 79% of maximal signal yield of UTE without fat suppression and is assumed more efficient with regard to signal yield in comparison to UTE with FatSat. Hopefully, further developments in WE-UTE will lead to optimization of a technique that can quickly and effectively visualize short T_2_ tissues like knee cartilage with positive and optimal contrast (80).

## Discussion

The suite of currently available quantitative UTE techniques has significantly improved the state of articular cartilage assessment, particularly for regions of cartilage with significant short T_2_ components such as the deep layer cartilage and calcified cartilage zone. This review first described the existing UTE sampling patterns, including radial, stack of spiral, and spiral, that have been utilized to study cartilage. In general, spiral sampling is more time-efficient than radial in covering the whole k-space because of its flexibility with regard to readout stretching. 3D sampling is a better choice for high-resolution cartilage imaging than 2D, especially for evaluation of the thin deep or calcified layers.

The review next systematically described the current state-of-the-art UTE techniques for assessment of cartilage, including both morphological and quantitative imaging. Morphological UTE techniques, including UTE echo subtraction, fat saturated T1-weghted UTE, UTE-DESS, IR, and Dual-IR prepared UTE, have been developed for high contrast imaging of the deep and calcified layers of cartilage. Generally, IR-based UTE sequences can generate a greater short T2 contrast compared to other non-IR-based techniques but require a much longer scan time. Many quantitative UTE imaging techniques have been developed including UTE-T_1_, UTE- ${\text{T}}_{2}^{*}$ , UTE-T_1ρ_, UTE-MT, UTE-CEST, and UTE-QSM. The biomarkers created by these techniques have different specificities: UTE-T_1_ is sensitive to the water content changes, UTE- ${\text{T}}_{2}^{*}$ is sensitive to the collagen integrity and calcification, UTE-T_1ρ_ is sensitive to the proteoglycan changes, UTE-MT is sensitive to collagen content and integrity, UTE-CEST is sensitive to the pH changes, and UTE-QSM is sensitive to susceptibility changes and calcification. Many of these biomarkers, such as UTE-T_1ρ_, UTE-MT and UTE-CEST, have been validated with both sample and patient studies in a number of promising studies. However, more comprehensive studies have yet to be performed that compare the performance of these biomarkers. We expect that more clinical trial studies could help answer the question.

The UTE biomarkers such as UTE-T_1ρ_ and ${\text{T}}_{2}^{*}$ , are limited by orientation sensitivity to the magic angle (57, 81, 82). Orientation-based changes may exceed the changes caused by degeneration in the cartilage itself. A few of the recently developed UTE techniques, such as UTE-AdiabT_1ρ_ and UTE-MT modeling, have shown promise in overcoming this so-called magic angle effect (57, 73, 74), suggesting that they have the potential to provide more robust evaluation of the cartilage composition and structural quality. Quantitative UTE biomarkers of MTR and MMF also exhibit feasibility to recognize enzymatic collagen degradation (83). Moreover, AdiabT_1ρ_ and T_1_ biomarkers have the capability to detect enzymatic proteoglycan (PG) loss in human knee cartilage (83).

There is only one study currently published in the literature that investigated the feasibility of simultaneous susceptibility mapping of the articular cartilage and cortical bone in knee (23). In this work, susceptibility variations were found in different layers of cartilage, which was in agreement with previous GRE-QSM studies (84, 85).

Fat signals shift radially in non-Cartesian UTE imaging due to the off-resonance sampling of fat. This significantly affects the deep and calcified layer cartilage imaging since these regions are close to the marrow fat region. Thus, fat suppression is important to reduce the fat signal contamination for both morphological and quantitative imaging. The conventional fat saturation technique is still the most widely used fat suppression technique in UTE, but the fat saturation pulse attenuates the cartilage signal due to direct and indirect saturation, especially for the shorter T_2_ layers. Newly developed fat saturation techniques such as the soft-hard water excitation pulse or single point Dixon method can avoid attenuating the cartilage signals while still performing effective fat suppression. Further studies to investigate the impact of quantitative UTE imaging for variable fat suppression techniques are highly valuable. In addition, field inhomogeneity can affect the effectiveness of fat suppression and generate off-resonance blurring. Furthermore, eddy currents can create an artificial tissue boundary, thus impacting the general brightness and contrast of UTE images. Therefore, accurate eddy correction is also critical for UTE imaging. Lastly, the integrity of high-resolution UTE imaging is of paramount importance for the accurate characterization of the thin structures of both the deep and calcified cartilage layers. Because the signal-to-noise ratio may be significantly reduced for high resolution imaging, a dedicated coil or deep learning-based denoise reconstruction should be applied to help improve the SNR performance of UTE imaging (32).

In addition to UTE MRI, a few other novel MRI techniques have been developed in recent years for qualitative and quantitative assessment of those tissues which have short T_2_ values (86). For example, zero echo time (ZTE) MRI, which utilizes a short rectangular excitation pulse during the fully ramped up readout gradient followed by fast radial sampling, is related to the UTE approach. Compared to UTE, ZTE has a shorter readout duration and therefore less ${\text{T}}_{2}^{*}$ contrast but also lacks flexibility in terms of ability to adjust either the field of view or flip angle. This makes each technique better suited for particular applications in the visualization of short T_2_ tissues (87). Larson et al. performed a comparative study between UTE and ZTE MRI techniques at 7T in the knee, ankle, and brain to assess differences between these two methods (48). They chose to use 7T field strength in order to take advantage of the increased polarization and also to accentuate any pulse sequence differences due to B_0_ and B_1_ inhomogeneities. This study observed that the UTE and ZTE techniques both achieved similar SNR efficiencies and image contrasts for volumetric imaging, but that UTE was more flexible in terms of volume selection, image contrast, and quantitative assessment of the tissue, and at a disadvantage in terms of sensitivity to gradient fidelity and higher acoustic noise.

The described MRI techniques in this review image hydrogen protons and proportions of water in various forms. However, sodium (i.e., Na^+^) and other elements have been of interest to researchers given that they may function as indicators of compositional changes in articular cartilage. Chang et al. performed a preliminary study of UTE and IR-UTE sodium MRI at 7T field strength on cartilage repair patients to investigate whether UTE MRI could detect sodium concentration in cartilage repair tissue, native cartilage adjacent to repaired tissue, and native cartilage on the side that was not involved in surgery (47). Significant differences between repair tissue and native tissue as well as between native tissues of different parts were identified by sodium IR-UTE. Cartilage Na^+^ concentration was found to be underestimated on non-fluid-suppressed MR imaging (sodium UTE).

The quantitative UTE MRI protocols described in this review usually require around 10 minutes of scan time, making them less optimal for clinical application given the heightened risk for motion artifacts. Therefore, for *in vivo* quantitative imaging, motion registration becomes a highly critical element of the protocol to limit artifacts resulting from patient movement during scans (88). Moreover, employing different accelerating techniques, such as stretching the readout trajectory (33), compressed sensing (89), integration of deep convolutional neural networks (CNNs) (32), 3D simplex deformable modeling (90), and attention U-Net with transfer learning, could and should be employed to accelerate the quantitative UTE protocols, largely reducing potential motion-related artifacts, with negligible errors. Interestingly, the U-Net with transfer learning framework has been applied for auto-segmentation of knee cartilage in quantitative UTE imaging (91).

## Conclusions

In this review, we have systematically introduced and discussed the major existing UTE MR techniques for assessment of knee cartilage. We expect that some of these UTE MRI techniques, especially for these magic angle-insensitive techniques, will be translated to clinical use in the near future to help improve diagnosis and treatment monitoring.

## Author Contributions

The conception and design of the study: AA and Y-JM. Acquisition of data: AA, SS, DM, GA, Y-JM, and JA. Writing-original draft preparation: AA and Y-JM. Writing-review and editing: Y-JM, AA, and HJ. Revising the manuscript critically for important intellectual content: Y-JM. Final approval of the version to be submitted: Y-JM and AA. All authors contributed to the article and approved the submitted version.

## Funding

The authors acknowledge grant support from NIH (R01AR078877, R01AR079484 and R21AR075851) and GE Healthcare. The authors declare that GE Healthcare was not involved in the study design, collection, analysis, interpretation of data, the writing of this article or the decision to submit it for publication.

## Conflict of Interest

GA was employed by the company BioSapien.

The remaining authors declare that the research was conducted in the absence of any commercial or financial relationships that could be construed as a potential conflict of interest.

## Publisher’s Note

All claims expressed in this article are solely those of the authors and do not necessarily represent those of their affiliated organizations, or those of the publisher, the editors and the reviewers. Any product that may be evaluated in this article, or claim that may be made by its manufacturer, is not guaranteed or endorsed by the publisher.
